# Nanomechanics of G-quadruplexes within the promoter of the *KIT* oncogene

**DOI:** 10.1093/nar/gkab079

**Published:** 2021-04-13

**Authors:** Enrico Buglione, Domenico Salerno, Claudia Adriana Marrano, Valeria Cassina, Guglielmo Vesco, Luca Nardo, Mauro Dacasto, Riccardo Rigo, Claudia Sissi, Francesco Mantegazza

**Affiliations:** School of Medicine and Surgery, BioNanoMedicine Center NANOMIB, University of Milano-Bicocca, 20854 Vedano al Lambro (MB), Italy; School of Medicine and Surgery, BioNanoMedicine Center NANOMIB, University of Milano-Bicocca, 20854 Vedano al Lambro (MB), Italy; School of Medicine and Surgery, BioNanoMedicine Center NANOMIB, University of Milano-Bicocca, 20854 Vedano al Lambro (MB), Italy; School of Medicine and Surgery, BioNanoMedicine Center NANOMIB, University of Milano-Bicocca, 20854 Vedano al Lambro (MB), Italy; School of Medicine and Surgery, BioNanoMedicine Center NANOMIB, University of Milano-Bicocca, 20854 Vedano al Lambro (MB), Italy; School of Medicine and Surgery, BioNanoMedicine Center NANOMIB, University of Milano-Bicocca, 20854 Vedano al Lambro (MB), Italy; Department of Comparative Biomedicine and Food Science, University of Padova, 35020 Legnaro (PD), Italy; Department of Pharmaceutical and Pharmacological Sciences, University of Padova, 35131 Padova (PD), Italy; Department of Pharmaceutical and Pharmacological Sciences, University of Padova, 35131 Padova (PD), Italy; Interdepartmental Research Center for Innovative Biotechnologies (CRIBI), University of Padova, 35121 Padova (PD), Italy; School of Medicine and Surgery, BioNanoMedicine Center NANOMIB, University of Milano-Bicocca, 20854 Vedano al Lambro (MB), Italy

## Abstract

G-quadruplexes (G4s) are tetrahelical DNA structures stabilized by four guanines paired via Hoogsteen hydrogen bonds into quartets. While their presence within eukaryotic DNA is known to play a key role in regulatory processes, their functional mechanisms are still under investigation. In the present work, we analysed the nanomechanical properties of three G4s present within the promoter of the *KIT* proto-oncogene from a single-molecule point of view through the use of magnetic tweezers (MTs). The study of DNA extension fluctuations under negative supercoiling allowed us to identify a characteristic fingerprint of G4 folding. We further analysed the energetic contribution of G4 to the double-strand denaturation process in the presence of negative supercoiling, and we observed a reduction in the energy required for strands separation.

## INTRODUCTION

G-Quadruplexes (G4s) are three-dimensional tetrahelical DNA structures consisting of stacks of planar guanine quartets occurring in G-rich regions of nucleic acid sequences (1). Different numbers of stacked quartets, the relative strand orientation and the loop composition are responsible for the polymorphism of G4s (2). A considerable number of such structures occur at the ends of chromosomes, where they are known to impair correct telomere maintenance (3,4). Moreover, a large number of G4s have been found at different genomic sites, where they play regulatory roles (5,6). Among these functions, a regulatory role of G4s in the transcriptional activity of several genes has been widely confirmed (7,8).

Recently, single-molecule (SM) techniques have been employed in the study of G4 conformations and nanomechanics (9–13). The advantage of the SM approach is that it allows a more specific analysis of the complex behaviour of G4s, which can be largely obscured by bulk techniques. In particular, force spectroscopy techniques allow the investigation of sample behaviours under mechanical stress conditions (14,15). Independent of the employed method, the majority of the currently available SM studies are focussed on the analysis of G4 unfolding in a single-stranded DNA context (16–21). The study of the nanomechanical properties of G4 embedded in ssDNA regions is appropriate for mimicking the biological situation of telomeric G4s, where there is a significant occurrence of G-rich DNA in a single-stranded form. However, in promoter regions, G4-forming sequences are inserted within the double helix, so the G4 structures can fold only upon bubble formation induced by enzymatic processes and/or torsional stress (22).

From an energetic point of view, the correlation between the presence of G4-forming sequences at certain genomic loci and the probability of bubble formation at those sites is still an open question. Indeed, it is not easy to answer this question via the application of standard ensemble techniques. In particular, in solution, mechanical stress cannot be maintained during thermal melting experiments, and factors such as the application of torque to dsDNA can considerably affect denaturation (23–25).

Among the available SM techniques, magnetic tweezers (MTs) allow the application of nanomechanical force and torque to single torsionally constrained dsDNA molecules to induce nanomechanical denaturation, which is a condition that can be considered similar to what is experienced by DNA during replication/transcription (26–31). Indeed, by imposing a specific twist to a torsionally constrained DNA and simultaneously applying a pulling force to the filament, it is possible to induce the opening of denaturation bubbles (32,33). If this nanomechanical denaturation involves a DNA region encompassing a G4-forming sequence, it will be feasible to explore the dsDNA-to-G4 transition. For the above reasons, MTs represents a powerful tool for studying the dynamics and nanomechanical characteristics of G4 formation in a context that mimics the non-telomeric configuration.

The usefulness of nanomechanics for studying G4 unfolding within dsDNA was first demonstrated by Selvam *et al.* (17), who applied magneto-optical tweezers to simultaneously impose a controlled twist and a pulling force in the tenths to hundreds of pN range on dsDNA. Similar to previous SM studies on ssDNA, experiments of Selvam *et al.* were based on the detection of the increase in DNA extension resulting from G4 unfolding events. Accordingly, by monitoring the impact of DNA torsional stress on G4 formation, the authors indirectly derived an increase in the G4 folding probability as a consequence of the application of negative supercoiling to the DNA (17).

The promoter of the *KIT* proto-oncogene, also known as CD117, encodes the mast/stem cell growth factor receptor c-kit. An increase in the expression of this gene is frequently associated with many forms of cancer, such as mastocytosis, gastrointestinal stromal tumours, lung cancer and leukemia (34). The regulation of *KIT* transcription levels appears to be largely dependent on the formation of three distinct G4 structures within the proximal promoter of the gene: kit2, kit* and kit1 (35,36). Accumulating evidence indicates that there is a reduction in *KIT* expression upon G4 formation at these sites (37,38). However, the molecular mechanisms leading to this result have not yet been fully elucidated. Indeed, whereas G4 formation might support the opening of the transcription bubble, the presence of G4s in the promoter might interfere with the proper recruitment of the transcriptional machinery (39). For this reason, it is relevant to understand how G4s form and their role in the modulation of DNA architecture.

In the present study, we analysed the G-quadruplex folding of the dsDNA domain of the *KIT* promoter, which comprises three G-rich sites, through the use of MTs under a low pulling force regime (<3 pN) over a large range of negative supercoiling. Under these conditions, the separation of the two DNA strands does not require the application of high force (>65 pN) (40) which would prevent G4 folding (23,32). The nanomechanical response of the *KIT* G4-forming domain (c-kit-wt) is compared to the behaviour of a dsDNA construct in which point mutations have been inserted to prevent the folding of the three G4s (c-kit-mut). In this context, we propose a new nanomechanical method for the detection of G4 formation that allows the simultaneous monitoring of G4 folding and quantification of the energy gain due to G4 formation for DNA bubble opening in the presence of the three G4s. MTs results indicate that, in spite of the higher GC content within the promoter region, the formation of G4s significantly decreases the double-strand helix stability; therefore, the total energy necessary for bubble formation is considerably lower in the presence of G4-forming sequences. This suggests a higher probability of G4 formation from ds-tracts than expected from the sequence composition and fits with the reported observation of G4s in cells during DNA processing (6).

## MATERIAL AND METHODS

### Preparation of DNA constructs

**Figure 1.**
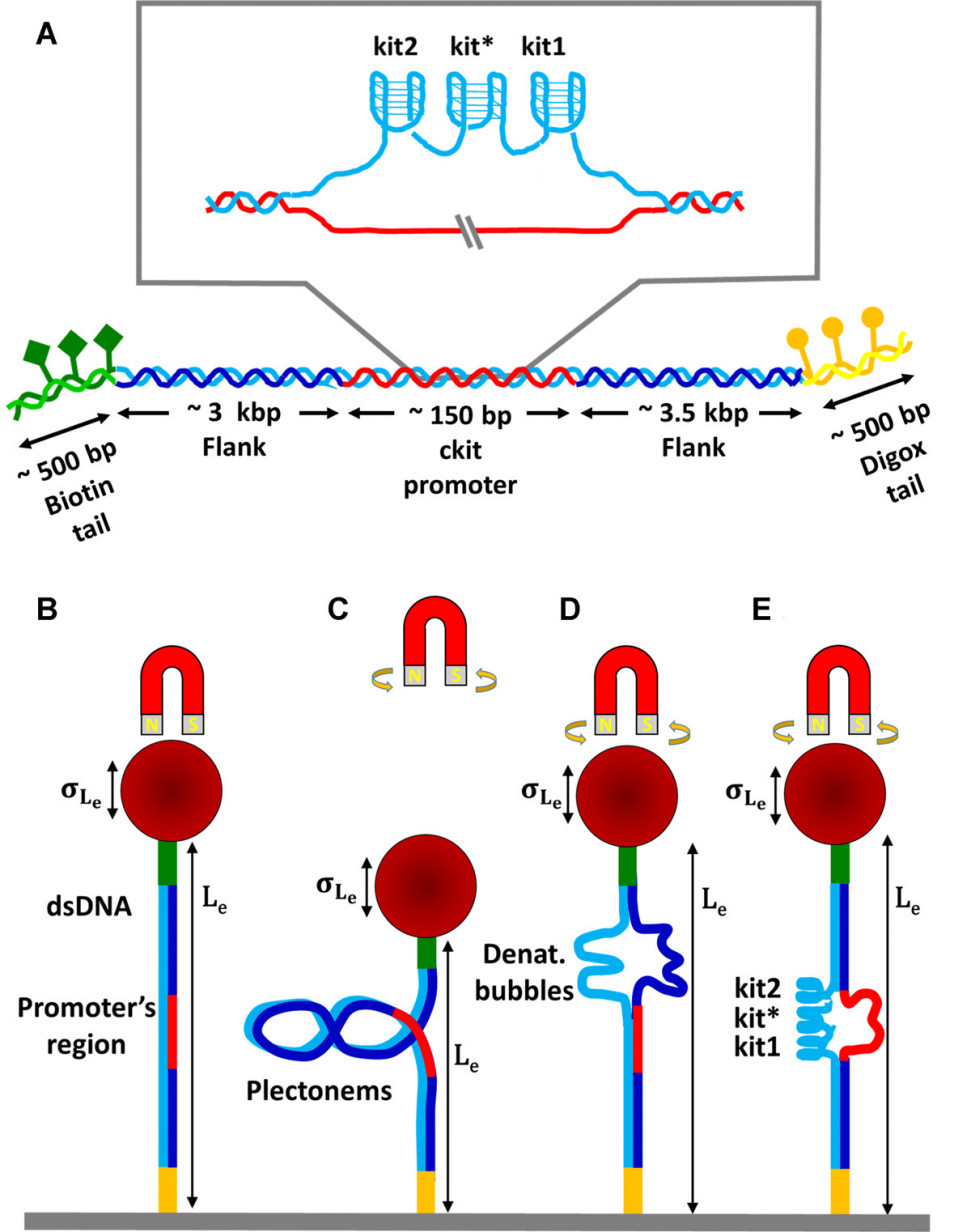
Simplified sketches of the investigated c-kit-wt construct and the MTs experiment strategy. (**A**) In the central core fragment (red and light blue filaments) containing the human *KIT* proximal promoter, three distinct G4s are embedded: kit2, kit* and kit1. This core fragment was flanked by two ∼3 kb flanking regions (dark and light blue filaments), which were enclosed by 5′ biotin- and 3′ digoxigenin-modified tails (green diamonds and yellow circles, respectively) to bind streptavidin-coated beads and an anti-digoxigenin functionalized microfluidic cell, respectively. (**B**–**E**) A para-magnetic bead (red sphere) is connected via dsDNA to the inner part of a microfluidic cell (grey stripe). By rotating the magnets, torque is applied to the DNA. By raising the magnets, the force applied to the DNA through the bead decreases, since the magnetic field decreases with the distance. In this condition, the formation of plectonemes instead of denaturation bubbles is favoured and the extension is reduced. In different states of torsion and force, the initial (B) DNA extension (L_e_) and its standard deviation (${\sigma _{{L_e}}}$) are modified as a consequence of plectonemes formation (C), the presence of denaturation bubbles (D) and G4 folding (E).

DNA tethers were constructed from five components: a 148 bp core fragment containing the *KIT* promoter region and two identical handles of 2881 bp, each flanking one side of the core fragment bookended by 3′ biotin- and 5′ digoxigenin-modified tails (see Figure 1A). In the case of the c-kit-wt construct, the G4-forming sequence corresponds to the portion between positions –158 and –87 upstream of the transcription starting site (TSS), which includes the three G4-forming sequences (in order from the 5′ end: kit2, kit* and kit1). A mutated form of the same sequence, referred to as c-kit-mut, in which each G4-forming sequence was subjected to point mutation to prevent G4 formation, was used as a negative control construct (see Supplementary Data for sequence details). The sequence of either c-kit-wt or c-kit-mut was inserted into the pGAL4.1 plasmid between the XmaI and NheI restriction sites.

The two flanking regions were obtained via mutagenic PCR amplification performed with LongAmp DNA polymerase (NEB) using Lambda DNA N6-methyladenine-free (Sigma-Aldrich, St. Louis, MO) as a template and the following forward primer: 5′-AAA**GGTAC***C****TC****GAG*TGCGACAGGTTTGATG-3′, and reverse primer: 5′-TTT**GGTAC***C****TC****GAG*CGAAATTAACTCTCAGG-3′. Each primer contains both XhoI (italic) and KpnI (underlined) restriction sites; the mutagenic regions are indicated in bold. The DNA sequence used as a template for the flanking region was chosen with online software because it was devoid of non-B DNA sequences (17, IDT Oligo Analyzer, https://eu.idtdna.com/pages/tools/oligoanalyzer).

Subsequently, the flanking regions were cloned into each plasmid upstream and downstream of the core sequence by taking advantage of the 3′ KpnI and the 5′ XhoI restriction sites, respectively. At the end of the two cloning steps, the 72 bp region encompassing the three G4-forming sequences was verified by a sequencing analysis, both in the case of the c-kit-wt and c-kit-mut plasmid. See Supplementary Data for details. To obtain a sufficient amount of the ‘flanking_core_flanking’ sequence, competent DH5α *Escherichia coli* strain cells were transformed with the plasmid containing either wt or mut insert. Plasmid DNA was extracted starting from a 50 ml cell culture that was grown overnight at 37°C using a Qiagen Midi Prep Kit. The purified DNA was then enzymatically digested with the ApaI and SacII restriction enzymes (NEB) by taking advantage of the two restriction sites already present in the pGAL4.1 plasmid and purified with a QIAquick PCR Purification Kit (Qiagen, Germantown, MD). The total length of the flanking core flanking sequence was 6680 bp.

The two functionalized tails were amplified by mutagenic PCR starting from the pBR322 plasmid (Roche, Basel, Switzerland) using Taq DNA polymerase (Euroclone, Italy). PCR was carried out in the presence of 20% biotin–16-dUTP- or 20% digoxigenin-11-dUTP-labeled nucleotides (Roche) using the forward mutagenic primers 5′-AAA**CCGCGG**CCAGAACATTTCTCTGGCCT-3′ and 5′-GCTT**GGGCCC**CAGAGTTCTTGAAGTGGTGG-3′, containing the SacII and ApaI restriction sites, respectively (underlined), and the same reverse primer: 5′-GGTCCAGTCGTCGGGTCTCGCGGTAT-3′.

The samples were purified by using the QIAquick PCR Purification Kit (Qiagen, Germantown, MD) and subsequently digested for 16 h using the appropriate restriction enzyme and subjected to further purification. Ligation between the two flanking-core-flanking sequences and the tails was carried out by taking advantage of the nick-joining activity of the T4 DNA ligase (NEB) at 16°C for 72 h. The final construct length was ∼7.5 kb. The presence of the final constructs was confirmed by 0.8% agarose gel electrophoresis.

### Magnetic tweezers setup and measurements

In this work, we used a custom MTs setup (41), essentially consisting of an inverted optical microscope (Nikon 60×, 1.49NA oil immersion with a 20-cm-focal-length tube lens) coupled with a system in which permanent neodymium magnets were located over a microfluidic flux cell (custom HybriWell3, Grace BioLabs, USA) for DNA incubation. Illumination was provided by an infrared (840 nm) superlumen diode (Superlum SLD-34-MP, UK) producing light endowed with high spatial coherence and low temporal coherence, allowing the focalization of the beam and achieving high intensity in the focal plane while avoiding out-of-focus interference. The images were acquired with a fast CMOS camera (EoSens 3CL Mikrotron) operating at 300 Hz with 1 MP of resolution and a 100 μs exposure time. The real-time image analysis allowed the simultaneous tracking of up to 40 beads over 3D with a resolution of 60 nm in the *x*–*y* plane and 10 nm in the *Z* direction.

The magnets could be translated along and rotated around the optical axis to control the tension and supercoiling of the DNA tethers. The inner surface of the microfluidic cell was functionalized overnight at 4°C with an anti-digoxigenin antibody at 20 μg/μl (Roche, Italy). The functionalized surface was then passivated overnight at 4°C with 10 mg/ml bovine serum albumin (Roche) in 150 mM phosphate-buffered saline, pH 7.4 (PBS). Streptavidin-coated superparamagnetic beads with a 1 μm diameter (Dynabeads MyOne Streptavidin C1, Dynal, Invitrogen, Milan, Italy) were tethered to the DNA filaments upon incubation at room temperature for 30 min. Finally, the bead-labelled DNA suspension was incubated in the microfluidic cell for 1 h before washing out the excess unbound tethers.

MTs measurements were carried out in Tris–HCl buffer supplemented with 0.1% Tween-20 and 0.1 mM EDTA added with KCl to promote G4 formation, or LiCl to inhibit G4 formation, or NaCl as control buffer. In all subsequent measurements, the KCl, LiCl, and NaCl concentrations were maintained at 150 mM.

### Force-extension curve and characteristic force

The outputs of a standard MTs experiment are the force extension and turn-extension curves, where the DNA end-to-end extension (*L*_e_) is measured as a function of either the applied force (*F*) or the number of imposed turns (*n*_t_), respectively (42–44). In a force extension curve, when no torsion is applied to the DNA (i.e. *n*_t_ = 0), *L*_e_ increases with F, reaching an asymptotic value. In this case, the curve is well described by the worm-like-chain (WLC) model (43,45), through the use of two fitting parameters: the DNA contour length (*L*_0_) and the DNA persistence length (*L*_P_) (46–48). When *n*_t_ is different from zero, the force extension curve exhibits a different shape with respect to the *n*_t_ = 0 situation (33). Under negative supercoiling (*n*_t_ < 0), the force extension curve presents two distinct regions: a low-force region, where the DNA extension is negligible, and a high-force asymptotic region, where the force extension curve is similar to that obtained in untwisted conditions (*n*_t_ = 0) and follows the WLC model. In the low-force regime, the filament is in a collapsed plectonemic state, while with increasing force, the imposed turns in the DNA are relaxed by opening denaturation bubbles, involving a number of base pairs proportional to the supercoil magnitude. These two regions are separated by a small range of forces at which the DNA extension value increases steeply between the collapsed and extended states, suggesting coexistence between plectonemes and denaturation bubbles (23,25). This DNA extension jump occurs at a characteristic force value (*F*_C_) depending on the DNA persistence length and base pairs melting energy (49). As a consequence of the coexistence of the two states, the fluctuations in DNA extension (evaluated on the basis of the variance $\sigma _{{L_e}}^2$ of the DNA extension) increase greatly in the transition region, and the *F* versus $\sigma _{{L_e}}^2$ plot clearly exhibits a bell-like shape, which allows the *F*_C_ at which $\sigma _{{L_e}}^2$ reaches its maximum value to be easily identified (24,50). Operatively, the detection of the characteristic force (*F*_C_) is obtained by acquiring several force–extension measurements at fixed imposed turns. The force ramp is performed by using an interval of 0.01 pN. For each force value, we considered the corresponding variance $\sigma _{{L_e}}^2$ of DNA extension. *F*_C_ is the force at which $\sigma _{{L_e}}^2$ reaches its maximum value.

## RESULTS

**Figure 2.**
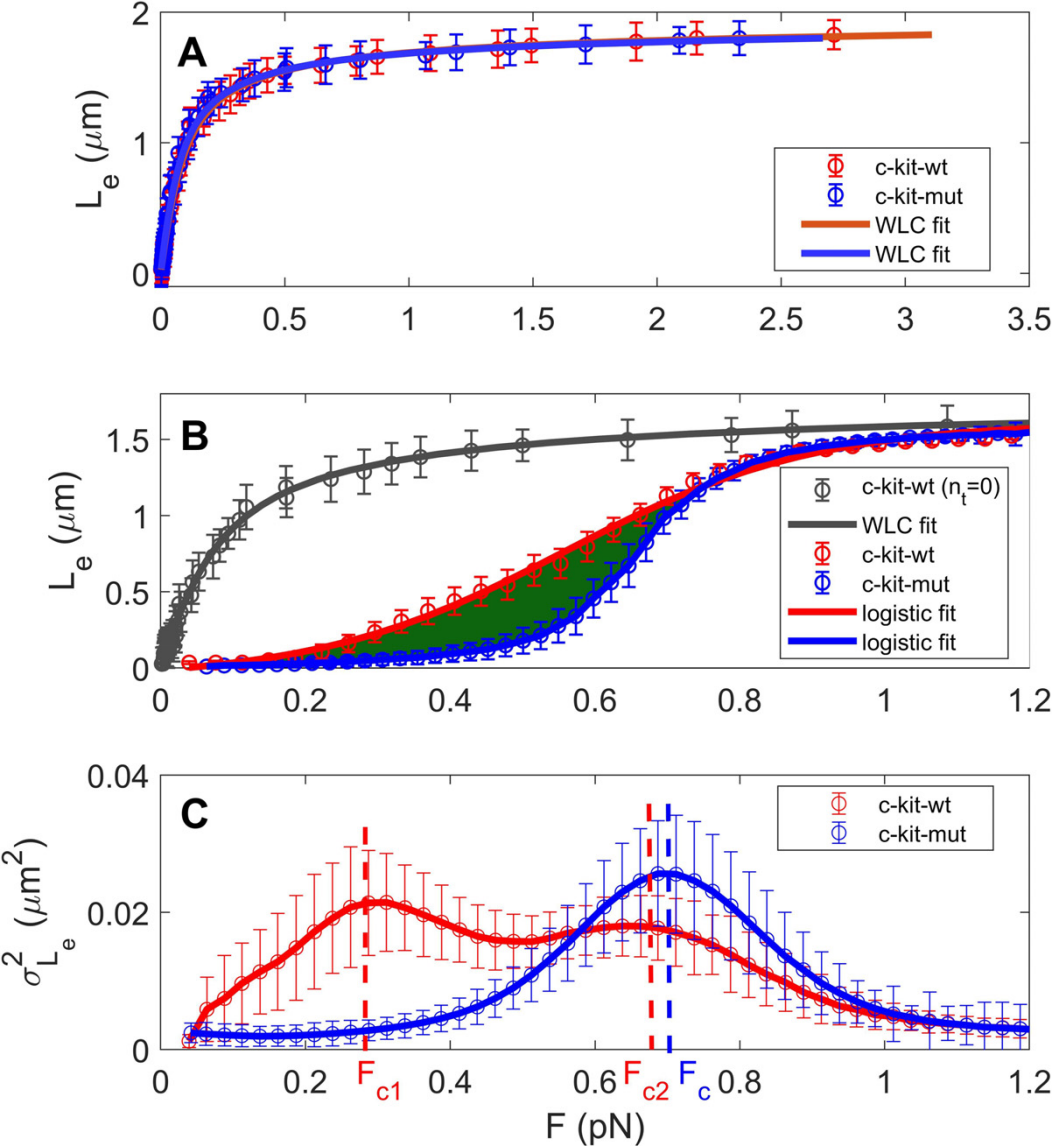
MTs-based nanomechanical characterization of the c-kit-wt (red) and c-kit-mut (blue) constructs at zero or negative supercoiling in 150 mM KCl. (**A**) Average force extension curves at *n*_t_ = 0, the standard deviations corresponding to every measurement are also reported. The continuous lines represent the best fit of the data to the WLC model (resulting free parameters: L_0_ = 2.0 ± 0.12 μm, L_P_ = 46 ± 4 nm for c-kit-wt and L_0_ = 1.97 ± 0.14 μm, L_P_ = 51 ± 5 nm for c-kit-mut). (**B**) Average force extension curves at *n*_t_ = –40. The error bars represent the standard deviations measured in hundreds of different force–extension curves. The continuous blue and red lines represent a fit to the logistic curve (Equation 1) of the data with fitting parameter values of *S*_0_ = 0.52 for c-kit-wt and *S*_0_ = 0.64 for c-kit-mut. The green area between the two curves corresponds to the energy difference between the two extension paths. (**C**) $\sigma _{{L_e}}^2$ variance of DNA extension measured as a function of the applied force (*F*) for a representative force extension curve at *n*_t_ = –40. The vertical dashed lines indicate the maximum value of $\sigma _{{L_e}}^2$ and the corresponding characteristic forces (*F*_C_ in the case of a single peak or *F*_C1_ and *F*_C2_ in the case of a double peak.

The investigated construct and the rationale for the MTs experiments are schematically represented in Figure 1, which illustrates how the formation of plectonemes (Figure 1C), DNA denaturation (Figure 1D), and G4 folding (Figure 1E) can affect the extension of DNA (*L*_e_) and its standard deviation (${\sigma _{{L_e}}}$) (see the Materials and Methods section for details about the constructs and the experimental setup). First, we tested whether the presence of G4s influences the response of the dsDNA filaments to nanomechanical stress by performing MTs force extension experiments with both the c-kit-wt and c-kit-mut constructs in different buffers (containing KCl, NaCl or LiCl at 150 mM, which promotes, has a neutral effect on, or inhibits G4 folding, respectively). Some representative measurements recorded in 150 mM KCl are shown in Figure 2 for both the wt and the mutant constructs. As shown in Figure 2A, at *n*_t_ = 0, there were no relevant differences in DNA extension measured as a function of the applied force between the two constructs. Namely, at *n*_t_ = 0, the *L*_e_ versus *F* data were well characterized by the WLC model (continuous lines in Figure 2A) with the same fitting parameters, independently of the presence of G4-forming sequences. Indeed, the resulting fit contour lengths were L_0_ = 2.00 μm ± 0.12 μm for c-kit-wt and L_0_ = 1.97 μm ± 0.14 μm for c-kit-mut, which are compatible with the number of base pairs (6680 bp excluding the functionalized tails) in the tethered DNA. The resulting fit persistence lengths provided similar results for c-kit-wt and c-kit-mut (*L*_P_ = 46 ± 4 nm and *L*_P_ = 51 ± 5 nm, respectively), in agreement with data reported for several DNA sequences (see Figure S2 and Table S1 in Supplementary Data for similar measurements in different buffers or different conditions) (51–53).

To identify nanomechanical fingerprints of G4 folding, we performed force extension experiments while imposing fixed negative torsional stress (*n*_t_ = –40 or supercoiling density σ = –40/(N_b_/10.4) = –0.070, where *N*_b_ is the number of base pairs) on the DNA molecule. Under these conditions, it is possible to explore the phase transition between the plectonemic and denatured states and to analyse the corresponding fluctuations in DNA extension (23–25). Representative force extension curves acquired for c-kit-wt and c-kit-mut under negative supercoiling (*n*_t_ = –40) in the presence of K^+^ ions are reported in Figure 2B. The value of *n*_t_ = –40 is within a range in which the imposed supercoiling allows the easy evaluation of the corresponding characteristic force. Under this torsional regime, in a relevant statistical ensemble, the force extension curves of c-kit-wt show less steep behaviour than those of c-kit-mut. Indeed, as is apparent from Figure 2B, low force (F ≈ 0.3 pN) is sufficient to extend c-kit-wt, while the same force did not increase the DNA extension of c-kit-mut, which remained negligible until a force *F* ≈ 0.5 pN. We also observed that at forces higher than 1 pN, the curves of c-kit-wt and c-kit-mut were superimposable, and they reached a common asymptotic value (*L*_0_) of DNA extension; the same *L*_0_ was obtained by applying the WLC model to the untwisted filaments (Figure 2A).

The observed nanomechanical difference between c-kit-wt and c-kit-mut is more evident considering the variance ($\sigma _{{L_e}}^2$) of the instantaneous DNA extension measured as a function of the applied force for *n*_t_ = -40. As illustrated in Figure 2C, under both the low force (<0.2 pN) and high force (>0.9 pN) regimes, where the DNA extension was almost zero or asymptotically *L*_0_ (respectively), the fluctuations of $\sigma _{{L_e}}^2$ were negligible. In contrast, under the intermediate regime of forces, the increment of DNA end-to-end extension was associated with an increase in $\sigma _{{L_e}}^2$. Precisely, the $\sigma _{{L_e}}^2$ versus *F* plots showed a single or double maximum. In particular, we observed that the majority of c-kit-mut molecules exhibited a single maximum of $\sigma _{{L_e}}^2$ at a characteristic force *F*_C_ ≈ 0.69 pN (see the representative blue curve in Figure 2C) as expected from previously reported experiments (23–25). In contrast, a relevant percentage of the c-kit-wt molecules show a double peak in the fluctuation of DNA extension under forces of *F*_C1_ and *F*_C2_, as shown by the representative red curve in Figure 2C.

Supplementary Figure S3 illustrates the results obtained by additional control experiments performed at *n*_t_ = –40 on c-kit-wt in different conditions (in the presence of 150 mM NaCl or LiCl). In all the cases, a small fraction of curves exhibits the behavior we associated with the formation of the G4 alternative secondary structure of DNA: a decrease in the steepness of the curve (*L*_e_ versus *F* plots) and a double peak distribution of the variance ($\sigma _{{L_e}}^2$versus *F* plots). These features differ in the occurrence but their characteristics are independent from the probability of the occurrence, for both the conditions tested.

We studied the dependence of the variance of the measured DNA extension as a function of the time window of acquisition or as a function of the applied force, on c-kit-wt in the presence of K^+^, used as reference (Supplementary Data, Figure S4). As shown in the figure, after a specific interval of time of about 3 s, $\sigma _{{L_e}}^2$ reaches an asymptotic value for all the forces explored. Accordingly, to ensure reliable results in the regimen of forces explored, all the measurements presented were acquired in a temporal window larger than 4 s.

**Figure 3.**
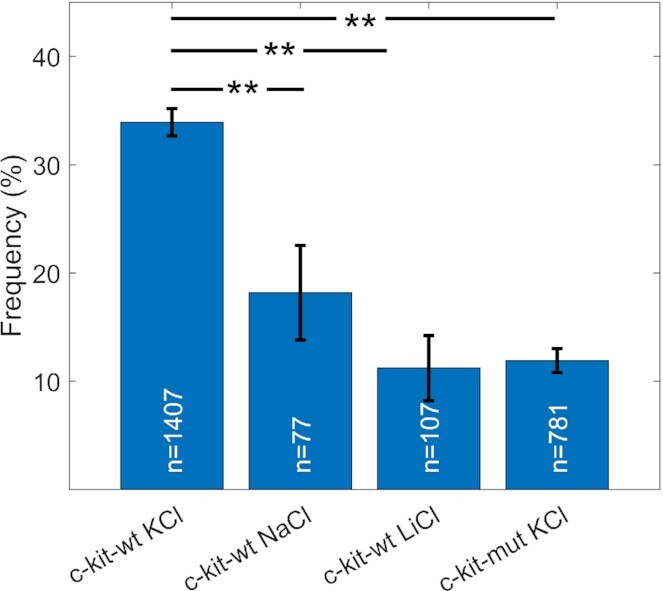
Percentage of double peaks in the $\sigma _{{L_e}}^2$ versus *F* curves obtained at *n*_t_ = −40 under different buffer conditions with two constructs: c-kit-wt in 150 mM KCl, 150 mM NaCl and 150 mM LiCl (first three columns); and c-kit-mut in 150 mM KCl (last column). The numbers (*n*) on the histogram bars indicate the total number of measurements acquired under the specific conditions. The error bars represent the corresponding standard deviations calculated assuming pure stochastic behaviour and considering the column height as the expected value and *n* as the sample size. The statistical analysis was performed with the ANOVA test. ** *P*< 0.001.

In Figure 3, we provide a detailed analysis of the statistical distribution of the presence of double peaks in the fluctuations of DNA extension. Specifically, on the first bar in Figure 3, we present the statistical percentage of double peak observations for c-kit-wt, as reported in the representative curves in Figure 2C. Here, the measurements were performed in the presence of K^+^ ions, a condition that promotes G4 folding due to the coordination of the cations with the stacked G-tetrads. To study whether the double peaks could be considered a nanomechanical fingerprint of G4 folding, the presence of single or double peaks in the fluctuations of $\sigma _{{L_e}}^2$ was investigated in three control samples: the c-kit-mut construct and c-kit-wt in different buffers (Figure 3) (see Figures S3 in Supplementary Data for $\sigma _{{L_e}}^2$ versus *F* data in other buffers).

In principle not only the variance, but also the DNA extension should be affected by the folding of the *KIT* G4s. However, this implies a modification of a few nm in the extension, which is difficult to be detected in the low force regimen. Conversely, in case of negatively imposed torsion, the opening of few bases leads to a large variance of the DNA extension due to the transition between the plectoneme formation and the opening of a denaturation bubble that completely changes the DNA arrangement. The G4s folding results detectable, since it significantly lowers the force of this transition.

In the presence of negative supercoiling, the DNA is under a stress condition, which needs to be relaxed. At low forces, the relaxation occurs by forming plectonemes, while at high forces, it occurs by forming denaturation bubbles (43). The formation of plectonemes reduces the DNA extension, conversely, the formation of denaturation bubbles does not severely influence the DNA extension. At intermediate forces, an increase in the fluctuations of DNA extension is observed. These fluctuations reach their maximum at a specific force value *F*_C_, which is interpreted, within our theoretical frame, as the equilibrium force between plectonemic and denaturation bubble conditions. Typically, a single peak in the $\sigma _{{L_e}}^2$ versus *F* plot is observed (24). The presence of a sequence able to form G4s in the conditions allowing its folding (i.e. in the presence of K^+^) opens the probability for a partial denaturation of the DNA aimed to reach this G4s state of single-strand folding. As a result, the presence of a sequence able to fold into G4s would decrease the energy necessary to open the DNA. This transition should occur at a force lower than *F*_C_. In the variance versus *F* plot, a second peak appears (at a *F* < *F*_C_), corresponding to the G4s formation, flanked by the typical peak at *F*_C_ ascribable to the plectonemes-to-denaturation bubbles transition, necessary to relax the overall torsion and reach the equilibrium.

As shown in Figure 3, the statistical percentage of double-peak observations depends on the presence of both the G4-forming sequence and the ions inducing G4 folding. Indeed, the experimental conditions known to promote G4 formation resulted in a double peak in the fluctuations of $\sigma _{{L_e}}^2$ more frequently than the conditions in which G4 formation was inhibited. In particular, among an ensemble of >2000 curves, double peaks were observed in ∼35% of the measurements of c-kit-wt in the presence of 150 mM KCl, which was the most efficient stabilizer of the G4 structure. This percentage decreased to ∼18% for measurements performed on c-kit-wt in the presence of the same concentration of NaCl, ∼11% for c-kit-wt in the presence of LiCl and ∼12% in the case of c-kit-mut in KCl buffer.

**Figure 4.**
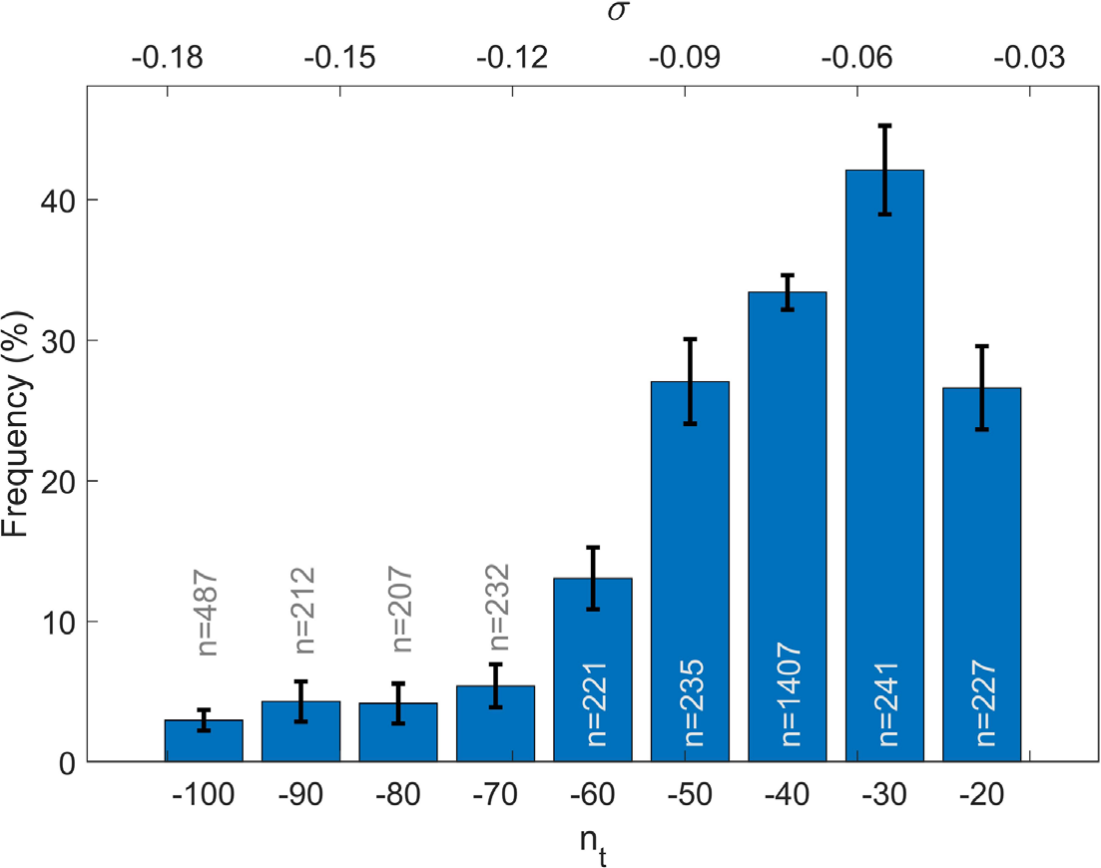
Percentage of double peaks in the fluctuations of DNA extension as a function of the imposed turns *n*_t_ (lower axes) or supercoiling density σ (upper axes). Data acquired for c-kit-wt in 150 mM KCl buffer. For each *n*_t_, the reported percentage was calculated for a number (*n*) of measurements, as indicated by the labels on the histogram columns. The error bars represent the corresponding standard deviations, calculated assuming purely stochastic behaviour and considering the column height as the expected value and *n* as the sample size.

To analyse whether the statistical distribution of double peaks was also influenced by the specific value of the imposed supercoiling, we studied the fluctuations in DNA extension at various *n*_t_ values. As shown in Figure 4, the percentage of the observed double peaks for c-kit-wt in 150 mM KCl reached a maximum at *n*_t_ = –30, while it decreased to <3% under a greater negative supercoiling regime (*n*_t_ = –100).

**Figure 5.**
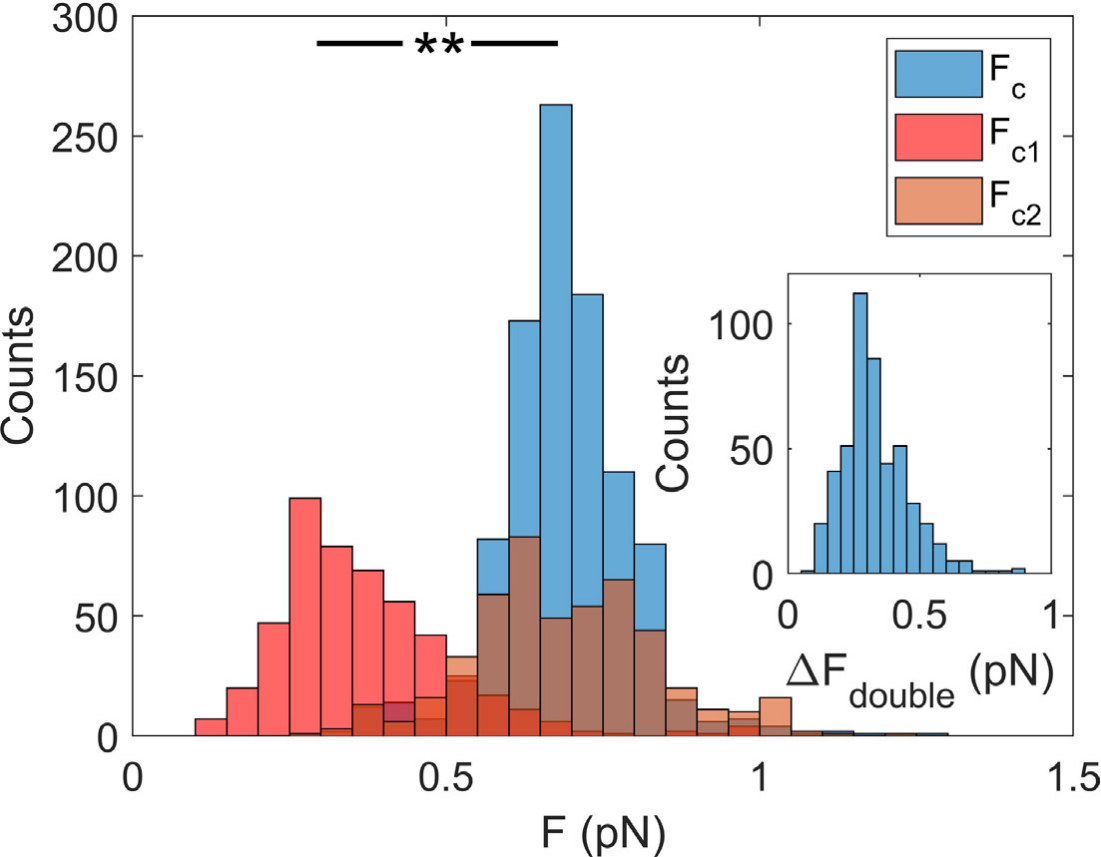
Characteristic force analysis. Statistical distribution of the measured values of the characteristic forces (*F*_C_, *F*_C1_, *F*_C2_) for c-kit-wt in 150 mM KCl. Single-peak characteristic force (*F*_C_) is indicated in blue, double peak characteristic forces in red (*F*_C1_) and orange (*F*_C2_). Data taken for *n*_t_ = –40, i.e. for supercoiling density σ = –0.070. Inset: statistical distribution of the force difference, Δ*F*_double_ = *F*_C1_ – *F*_C2_. The statistical analysis was performed with the *T* test. ** *P*< 0.001.

In Figure 5, we report the statistical distribution of the characteristic force values measured for *n*_t_ = –40, i.e. for supercoiling density *σ* = –0.070, in the case of both a single peak (*F*_C_) and a double peak (*F*_C1_ and *F*_C2_). In the latter case, G4 folding induces a local maximum in the fluctuations at a lower force value (*F*_C1_ = 0.37 ± 0.14 pN), while a higher characteristic force (*F*_C2_ = 0.69 ± 0.15 pN) coincides with the characteristic force observed in the curves with a single peak (*F*_C_ = 0.69 ± 0.11 pN). In the inset of Figure 5, the statistical distribution of Δ*F*_double_ (i.e. the difference between *F*_C1_ and the corresponding *F*_C2_ (Δ*F*_double_ = *F*_C2_ – *F*_C1_)) confirms the systematic difference between the two characteristic force values.

**Figure 6.**
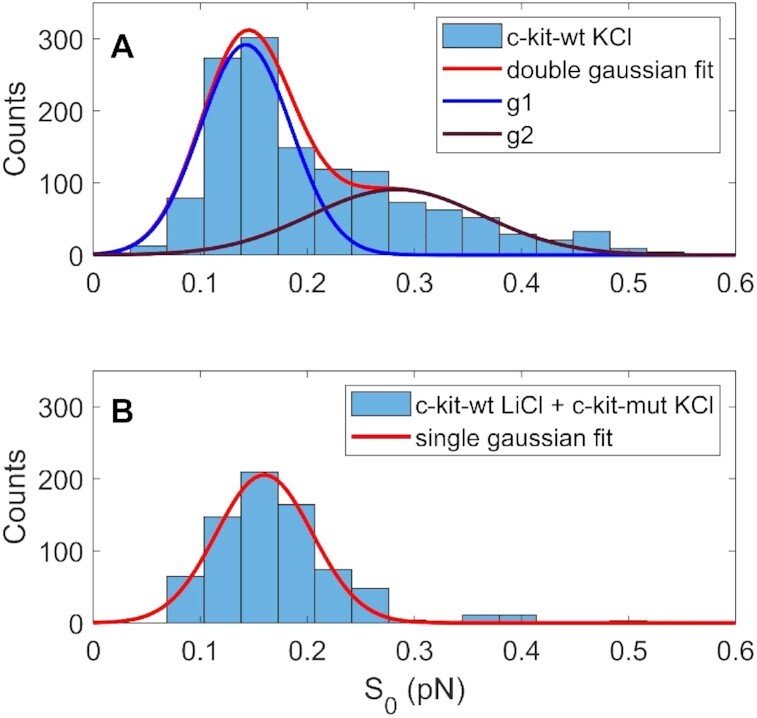
Statistical distribution of the steepness parameter (*S*_0_) derived from the fitting of the *L*_e_ versus *F* curves with the logistic function (Equation 1). Data acquired *n*_t_ = –40 for c-kit-wt in KCl (A) and for c-kit-wt in LiCl and c-kit-mut in KCl (B). The continuous lines represent the fit of the reported data to a single Gaussian (red line in panel (**B**)) or to a double Gaussian (red line of panel (**A**) resulting from the sum of the single Gaussians described by the blue (g1) and purple (g2) curves). The Wilcoxon test shows *P*< 0.001, confirming the significant difference between the two distributions described in panels (A) and (B).

Finally, since the *L*_e_ versus *F* curves corresponding to $\sigma _{{L_e}}^2$ versus *F* curves exhibiting a single peak present on average a higher steepness compared to those measured for double-peaked $\sigma _{{L_e}}^2$ versus *F* curves, we quantified this feature by fitting the data with a phenomenological logistic function given by the following expression:

(1) $$\begin{equation*}{L_e} = \ a + \frac{b}{{1 + {e^{\frac{{F - F1}}{{{S_0}}}}}}}\end{equation*}$$

where *a* and *b* are two normalization constants, *S*_0_ is a parameter that represents the curve steepness and *F*_1_ is the symmetric point of the logistic function. The statistical distributions of the *S*_0_ parameter for c-kit-wt and c-kit-mut were significantly different, as verified by the Wilcoxon test, (*P* < 0.001 data shown in Figure 6).

It is worth noting that analogous measurements of *L*_e_ and $\sigma _{{L_e}}^2$ as a function of the applied force were performed at positive turns. In the various samples, no relevant differences were found between the condition in which the G4 structures are induced or prevented to form (see Figure S5 in Supplementary Data). In addition, for every condition tested, we never recorded any double peak in the $\sigma _{{L_e}}^2$versus *F* plot. These data confirm what already observed by Marko and Neukirch: in the positive supercoiling regimen, for *n*_t_ > 0 the DNA phase transitions does not involve the dsDNA opening necessary for the G4 folding (32).

## DISCUSSION

In this work, the MTs technique was applied to analyse the nanomechanical characteristics of a domain of the proximal promoter of the *KIT* proto-oncogene that contains three G4-forming sites. We observed that, in the absence of supercoiling, the presence of these G4-forming sequences did not alter the nanomechanical properties of the double-stranded DNA filament. Indeed, the measured standard nanomechanical parameters, such as contour and persistence lengths, were not affected by the presence of the G4 sequences. These results appear reasonable, given that such parameters are normally weakly dependent on the specific DNA sequence composition (54). Furthermore, the number of bases involved in the three distinct G4s within the *KIT* promoter represents ∼2% of the total number of bases in the DNA filament. However, the presence of these few bases in the *KIT* promoter sequence were revealed when the DNA filament was under the effect of negative torsion.

In general, the folding of a G4 structure in a dsDNA filament needs to be preceded by the separation of the two strands. In vitro, strand opening can be obtained in the absence of negative torsion by applying force exceeding 65 pN (40). However, the DNA extension imposed by such high forces would prevent G4 folding. In contrast, in the presence of negative supercoiling, nanomechanical denaturation can be obtained at forces below 1 pN (23,32) under which a G4 structure can easily fold.

In this negative supercoiling regime, a peculiar nanomechanical behaviour was observed for the DNA sequence under investigation; i.e., a double peak occurred in the $\sigma _{{L_e}}^2$ versus *F* curve. In the presence of K^+^, which is an ion favouring G4 folding, we observed a statistically significant increase in the percentage of double peaks for the DNA containing the c-kit-wt promoter with respect to the controls, in which either mutations or different ions prevent G4 folding. In addition, we observed that the occurrence of the double peak recapitulated the efficacy of the different ions in coordinating and stabilizing G4 structures as described in the literature: K^+^> Na^+^> Li^+^ (55,56). Indeed, the percentage of double peaks, which was 35% in association with K^+^, was almost halved, to 18%, in the presence of Na^+^ and was further reduced to 11% in association with Li^+^. The statistical correlation between the appearance of a double peak in the $\sigma _{{L_e}}^2$ versus *F* curve and conditions favouring or inhibiting G4 folding suggests that the occurrence of the double peak might be considered as a fingerprint of G4 formation. Moreover, the statistical distribution of double peaks observed under the different experimental conditions was in qualitative agreement with the probability distribution of G4 formation at the single-molecule level, measured by monitoring the unfolding events occurring under high tension (∼40 pN) by Selvam and colleagues (17). Since we studied a different promoter, only a qualitative comparison with their work can be performed. Nevertheless, the discrepancy between the occurrence of G4s in c-kit-mut in K^+^ (12%) and c-kit-wt in Li^+^ (11%) indicated by our data and the corresponding values (3% and 4%, respectively) presented in Selvam's work deserves a comment. Regarding the data obtained in the presence of K^+^, this discrepancy could be reasonably due to the different characteristics of the control samples. In Selvam's paper, the sequence used as a control did not contain the G4 sequence, while in our case, the c-kit-mut contained only a few mutated residues; thus, despite the inserted point mutations, some weakly folded states may still occur (57–59). Furthermore, as highlighted above, the promoter sequence analysed in our experiments differed from that considered in Selvam's work. In particular, we studied a longer sequence containing three distinct sub-regions that could fold into G4s either independently or through G4–G4 cross-talk (60,61). Such a long sequence is more prone to present alternative single-strand structures (i.e. hairpins or incompletely paired domains, which may interfere with G4 folding recognition).

We cannot exclude that the residual double peaks observed in the negative controls (about 12% instead of 0%) can be related to the formation of alternative secondary structures (e.g. hairpin or cruciform extrusions) able to relax the negative turns imposed to the DNA, with an energy gain comparable to the one we attribute to the G4s (62,63). Anyway, a statistically significant increment in this percentage is observed in the conditions favouring the G4s formation. On the other side, the probability of double peaks occurrence (∼35%) could depend on the denaturation bubble opening, which is a random event: in all the cases in which plectonemes hide the *KIT* promoter region, the G4s formation is prevented, reducing the probability of double peaks occurrence. Moreover, the DNA geometry in negative supercoiling could also contribute by partially preventing G4s formation.

Our results suggest that the presence of a G4-forming sequence alters the energetic balance between plectonemes formation and bubble opening. Indeed, for *n*_t_ < 0, the presence of a low-force secondary peak at *F*_C1_ = 0.37 pN in the $\sigma _{{L_e}}^2$ versus *F* curves suggests that the transition between bubbles and plectonemes formation is anticipated at lower force values. In addition, it was possible to quantify the energetic contribution of the G4 structure by using the following equation, which expresses the energy cost α of relaxing a single turn by opening the double strand in terms of the characteristic force (*F*_C_) and bending constant (*B=L_P_k_B_T*) where *k_B_* is the Boltzmann constant and *T* is the temperature (23,25):

(2) $$\begin{equation*}\alpha \ = \ \pi \sqrt {8B{F_C}} \end{equation*}$$

If we ascribe a characteristic force of *F*_C1_ = 0.37 pN to the DNA transition between double-strand and G4 folded structures and a characteristic force of *F*_C2_ = 0.69 pN to the transition between the double-strand and denaturation bubble structures, we can calculate the ratio between the two transition energies by using the following formula:

(3) $$\begin{equation*}\sqrt {\frac{{{F_{C1}}}}{{{F_{C2}}}}} = \ 0.73\end{equation*}$$

Considering the whole G4-forming sequence present in the DNA construct (see Supplementary Data), we expect an energy cost of approximately 120 kcal/mol will be incurred in opening the G4-forming sequence (54,64). This value, multiplied by the calculated factor of 0.73, results in a cost of 88 kcal/mol in the case of G4 formation, with a net energy gain of 32 kcal/mol. This result is close to the expected energy gain calculated by assuming a value of 10 kcal/mol, predicted for each G4 formation (54,65), and by considering the presence of three G4s in the investigated sequence.

Since the melting of the double strand corresponds to an increase in filament extension, the occurrence of partial denaturation of c-kit-wt in K^+^ under low force indicates early destabilization of the double helix, which is also observed in the force extension curve as a decrease in the steepness of the *L*_e_ vs *F* curves under negative supercoiling. These data show that the DNA filament in the presence of G4 sequences is more easily extended under lower forces, i.e. the plectonemic structure is less probable than denaturation bubbles. As a result, the statistical distribution of the *S*_0_ parameter obtained from the logistic fit, which describes the steepness of the force extension curve, is considerably different for c-kit-wt in K^+^ (high probability of G4 folding) than for c-kit-mut in K^+^ and c-kit-wt in Li^+^ (low probability of G4 folding). Even though the superposition area of the two Gaussian distributions shown in Figure 6A is too large to clearly identify a single G4 folding event by using only the steepness parameter (*S*_0_), a difference in the shape of the two distributions can be observed. In particular, for the control sequences, a single Gaussian fit describes the *S*_0_ distribution well. In contrast, the statistics for c-kit-wt in K^+^ are described by a double Gaussian distribution, and the calculated relative Gaussian areas are 63% for the high-steepness Gaussian (lower S_0_) and 37% for the low-steepness Gaussian (high *S*_0_), in good agreement with the relative abundance of the double peak (35%, as shown in Figure 3).

The analysis of the force–extension curves in the presence of negative supercoiling confirmed the ability of the MTs technique to reveal G4 folding events. It also provided additional information, since it enabled the quantification of the energetic contribution in the destabilization of the double helix due to the presence of the *KIT* promoter sequence. A comparison between the mean force extension curves related to the double-peak cohort and the single-peak cohort revealed a significant difference in the work of the magnetic pulling force exerted to extend the DNA. In fact, the work applied to extend the DNA in the case of the curves showing a double peak of $\sigma _{{L_e}}^2$ was significantly lower than the work applied in the case of a single peak. This difference could be easily calculated by measuring the area between the two curves (green area in Figure 2B), resulting in a value of 24 ± 6 kcal/mol, which is in reasonable agreement with the value calculated using the formula extracted from a published mechanical model (see Equation 2) (23) and compatible with the expected energy gain due to G4 formation (54,64). These three converging estimates suggest that the proposed approach is valuable and can provide reliable quantification of the energetic change in double-strand stability induced by the presence of G4 sequences under realistic conditions of a negative torque and pulling force close to physiological conditions.

A further indication of the correlation between G4 formation and double-peak occurrence emerges from Figure 5, where the dependence of the statistical distribution of the double peak is reported as a function of the applied supercoiling. Indeed, the data suggest the existence of specific negative torsion that is more efficient in promoting G4 folding. This negative value of *n*_t_ around 30 turns implies the opening of ∼100 bp region of a DNA sequence of 6680 bp, compatible (23) with the length of the *KIT* promoter sequence, which is 72 bp long.

In principle, it would be possible to identify different substructures (folding of single or double G4s only) that could be folded within the promoter sequence. Actually, the histogram relative to the distribution of forces reported in Figure 5 shows a large single peak distribution (red histogram) in the low force regime, which can be supposed to be due to the presence of multiple peaks summed up. Unfortunately, this hypothesis cannot be verified by analysing our data, since the intrinsic noise due to the Brownian motion of the bead limits the experimental resolution, hindering the discrimination between a single wide peak and an ensemble of multiple narrow peaks close one to the other.

Overall, we can conclude that the presence of G4-forming sequences induces a counterintuitive alteration of nanomechanical DNA behaviour. Indeed, despite the presence of a large number of GC base pairs, characterized by more stable pairing (54), DNA containing G4 sequences is more easily locally denatured under suitable torsion. Indeed, since the local separation of the double strand is necessary before G4 folding, the formation of these DNA secondary structures is energetically facilitated by negative torsion. According to the analysis of the force extension curves, the energy gain in this step derived from the formation of three G4s can be estimated to be ∼24 kcal/mol.

Thus, our findings suggest that the G4-forming sequences within the *KIT* promoter are crucial in determining the overall architecture of the promoter itself and, thus, contribute to the dynamic fine regulation of gene expression. Therefore, our results further suggest that the G4s within the *KIT* promoter could represent successful candidates for targeted therapy in the case of the misregulation of this proto-oncogene.

## Supplementary Material

gkab079_Supplemental_File

## Notes

Present address: Luca Nardo, Department of Science and High Technology, University of Insubria, 22100 Como (CO), Italy.

Present address: Guglielmo Vesco, Department of Science and High Technology, University of Insubria, 22100 Como (CO), Italy.
