# Supplementary material for: Nanomechanics of G-quadruplexes within the promoter of the KIT oncogene
Source: Nucleic Acids Res. 2021 Apr 13;49(8):4564–73. doi: 10.1093/nar/gkab079 (PMC8096272; doi:10.1093/nar/gkab079)
Supplement: gkab079_Supplemental_File [file gkab079_supplemental_file.pdf]

Nanomechanics of G-quadruplexes within the promoter of the *c-kit* oncogene.

Enrico Buglione[1], Domenico Salerno[1,*], Claudia Adriana Marrano[1], Valeria Cassina[1], Guglielmo Vesco[1], Luca Nardo[1], Mauro Dacasto[2], Riccardo Rigo[3], Claudia Sissi[3,4], Francesco Mantegazza[1]

[1] School of Medicine and Surgery, NANOMIB Nanomedicine Center, University of Milano-Bicocca, 20854 Vedano al Lambro (MB), Italy.
[2] Department of Comparative Biomedicine and Food Science, University of Padova, 35020 Legnaro (PD), Italy.
[3] Department of Pharmaceutical and Pharmacological Sciences, University of Padova, 35131 Padova (PD), Italy.
[4] Interdepartmental Research Center for Innovative Biotechnologies (CRIBI), University of Padova, 35121 Padova (PD), Italy.

SUPPLEMENTARY DATA

Sequencing analysis of the *c-kit-wt* and *c-kit-mut* plasmids

After the cloning step of the flanking-region, the plasmids encompassing the c-kit-wt and c-kit-mut construct were both verified by sequencing using two different primers: cKIT_seqFW, a forward primer annealing in 5’ to the c-kit promoter region and the cKIT_seqREV, a reverse complement primer annealing in 3’ to the c-kit promoter region.

Primers

```
cKIT_seqFW       5′  AGACGCCGCCGGGAAGAA  3′
cKIT_seqREV      5′  TAGCGCGGCAAAGCCGAG  3′
```

The sequencing outputs and the multiple alignments with the 72 bp-sequence expected for c-kit-wt (c-kit exp), obtained with the online *CLUSTAL Ω (1.2.4) multiple sequence alignment tool (EMBL-EBI, https://www.ebi.ac.uk/Tools/msa/clustalo/)*, are reported hereafter.

Sequencing outputs

>c-kit-wt FW
AGGCGAGGAGGGGCGTGGCCGGCGCGCAGAGGGAGGGCGCTGGGAGGAGGGGCTGCTGCTCGCCGCTCGC
GGCTCTGGGGGCTCGGCTTTGCCGCGCTAGCCTCGAGCGAAATTAACTCTCAGGCACTGCGTGAAGCGGC
AGAGCAGGCAATGCATGACGACTGGGGATTTGACGCAGACCTTTTCCATGAATTGGTAACACCATCGATT
GTGCTGGAACTGCTGGATGAACGGGAAAGAAACCAGCAATACATCAAACGCCGCGACCAGGAGAACGAGG
ATATTGCGCTAACAGTAGGGAAACTGCGTGTTGAGCTTGAAACAGCAAAATCAAAACTCAACGAGCAGCG
TGAGTATTACGAAGGTGTTATCTCGGATGGGAGTAAGCGTATTGCTAAACTGGAAAGCAACGAAGTCCGT
GAAGACGGAAACCAGTTTCTTGTTGTTCGCCATCCTGGGAAGACTCCTGTTATCAAGCACTGCACTGGTG
ACCTGGAAGAGTTTCTGCGGCAGTTAATCGAACAAGACCCGTTAGTAACTATCGACATCATTACGCATCG
CTATTACGGGGTTGGAGGTCAATGGGTTCAGGATGCAGGTGAGTATCTGCATATGATGTCTGACGCTGGC
ATTCGCATCAAAGGAGAGTGAGATCGGTTTTGTAAGAGATAACGCTTGTGAAAATGCTGAATTTCGCGTC
GTCTTCACAGCGATGCCAGAGTCTGTAGTGTCAGATGATGACCGTACTCAAACATCGGGTTGAGTATTAT
CTTACTGTTTCTTTACATAAACATTGCTGATACCGTTTAGCTGAAACGACATACATTGCAAGGAGTTTAT
AAATGAGTATCAATGAGTTAGAGTCTGAGCAAAAAGATTGGGCGTTATCAATGTTGTGCAGATCCGGTGT
CTTGTCTCCATGCAGACATCACGAAGGTGTTTATGTAGATGAAGGTATAGATATAGAGTCGGCATACAA

>c-kit-wt REV COMP
CGAGCAGCAGCCCCTCCTCCCAGCGCCCTCCCTCTGCGCGCCGGCCACGCCCCTCCTCGCCTCCCCTCCC
TCGCGCCCGCCCGGGTCTCGCTTCTTCCCGGCGGCGTCTGGTACCTCGAGTGCGACAGGTTTGATGACAA
AAAATTAGCGCAAGAAGACAAAAATCACCTTGCGCTAATGCTCTGTTACAGGTCACTAATACCATCTAAG
TAGTTGATTCATAGTGACTGCATATGTTGTGTTTTACAGTATTATGTAGTCTGTTTTTTATGCAAAATCT
AATTTAATATATTGATATTTATATCATTTTACGTTTCTCGTTCAGCTTTTTTATACTAAGTTGGCATTAT
AAAAAGGCATTGCTTATCAATTTGTTGCAACGAACAGGTCACTATCAGTCAAAATAAAATCATTATTTGA
TTTCAATTTTGTCCCACTCCCTGCCTCTGTCATCACGATACTGTGATGCCATGGTGTCCGACTTATGCCC
GAGAAGATGTTGAGCAAACTTATCGCTTATCTGCCTCTCATAGAGTCTTGCAGACAAACTGCGCAACTCG
TGAAAGGTAGGCGGATCCCCTTCGAAGGAAAGACCTGATGCTTTTCGTGCGCGCATAAAATACCCTGATA
CTGTGCCGGATGAAAGCGGTTCGCGACGAGTAGATGCAATTATGGTTTCTCCGCCAAGAATCTCTTTGCA
TTTATCAAGTGTTTCCTTCATTGATATTCCGAGAGCATCAATATGCAATGCTGTTGGGATGGCAATTTTT
ACGCCTGTTTTGCTTTGCTCGACATAAAGATATCCATCTACGATATCAGACCACTTCATTTCGCATAAAT
CACCAACTCGTTGCCCGGTAACAACAGCCAGTTCCATTGCAAGTCTGAGCCAACATGGTGATGATTCTGC
TGCTTGATAAATTTTCAGGTATTCGTCAGCCGTAAGTCTTGATCTCCTTACCTCTGATTTTGCTGCGCGA
G

>c-kit-mut FW
GAGTCGATGAGGGGCGTGGCCGGCGCGCAGAGGGAGTTCGCTGGGAGGAGGGGCTGCTGCTCGCCGCTCG
CGGCTCTGGGGGCTCGGCTTTGCCGCGCTAGCCTCGAGCGAAATTAACTCTCAGGCACTGCGTGAAGCGG
CAGAGCAGGCAATGCATGACGACTGGGGATTTGACGCAGACCTTTTCCATGAATTGGTAACACCATCGAT
TGTGCTGGAACTGCTGGATGAACGGGAAAGAAACCAGCAATACATCAAACGCCGCGACCAGGAGAACGAG
GATATTGCGCTAACAGTAGGGAAACTGCGTGTTGAGCTTGAAACAGCAAAATCAAAACTCAACGAGCAGC
GTGAGTATTACGAGGGTGTTATCTCGGATGGGAGTAAGCGTATTGCTAAACTGGAAAGCAACGAAGTCCG
TGAAGACGGAAACCAGTTTCTTGTTGTTCGCCATCCTGGGAAGACTCCTGTTATCAAGCACTGCACTGGT
GACCTGGAAGAGTTTCTGCGGCAGTTAATCGAACAAGACCCGTTAGTAACTATCGACATCATTACGCATC
GCTATTACGGGGTTGGAGGTCAATGGGTTCAGGATGCAGGTGAGTATCTGCATATGATGTCTGACGCTGG
CATTCGCATCAAAGGAGAGTGAGATCGGTTTTGTAAAAGATAACGCTTGTGAAAATGCTGAATTTCGCGT
CGTCTTCACAGCGATGCCAGAGTCTGTAGTGTCAGATGATGACCGTACTCAAACATCGGGTTGAGTATTA
TCTTACTGTTTCTTTACATAAACATTGCTGATACCGTTTAGCTGAAACGACATACATTGCAAGGAGTTTA
TAAATGAGTATCAATGAGTTAGAGTCTGAGCAAAAAGATTGGGCGTTATCAATGTTGTGCAGATCCGGTG
TCTTGTCTCCATGCAGACATCACGAAGGTGTTTATGTAGATGAAGGTATAGATATAGAGTCGGCATACAA
A

>c-kit-mut REV COMP

```
CGAGCAGCAGCCCCTCCTCCCAGCGAACTCCCTCTGCGCGCCGGCCACGCCCCTCATCGACTCCCCTCCC
TCGCGCACGCACGGGTCTCGCTTCTTCCCGGCGGCGTCTGGTACCTCGAGTGCGACAGGTTTGATGACAA
AAAATTAGCGCAAGAAGACAAAAATCACCTTGCGCTAAAGCTCTGTTACAGGTCACTAATACCATCTAAG
TAGTTGATTCATAGTGACTGCATATGTTGTGTTTTACAGTATTATGTAGTCTGTTTTTTATGCAAAATCT
AATTTAATATATTGATATTTATATCATTTTACGTTTCTCGTTCAGCTTTTTTATACTAAGTTGGCATTAT
AAAAAAGCATTGCTTATCAATTTGTTGCAACGAACAGGTCACTATCAGTCAAAATAAAATCATTATTTGA
TTTCAATTTTGTCCCACTCCCTGCCTCTGTCATCACGATACTGTGATGCCATGGTGTCCGACTTATGCCC
GAGAAGATGTTGAGCAAACTTATCGCTTATCTGCTTCTCATAGAGTCTTGCAGACAAACTGCGCAACTCG
TGAAAGGTAGGCGGATCCCCTTCGAAGGAAAGACCTGATGCTTTTCGTGCGCGCATAAAATACCCTGATA
CTGTGCCGGATGAAAGCGGTTCGCGACGAGTAGATGCAATTATGGTTTCTCCGCCAAGAATCTCTTTGCA
TTTATCAAGTGTTTCCTTCATTGATATTCCGAGAGCATCAATATGCAATGCTGTTGGGATGGCAATTTTT
ACGCCTGTTTTGCTTTGCTCGACATAAAAATATCCATCTACGATATCAGACCACTTCATTTCGCATAAAT
CACCAACTCGTTGCCCGGTAACAACAGCCAGTTCCATTGCAAGTCTGAGCCAACATGGTGATGATTCTGC
TGCTTGATAAATTTTCAGGTATTCGTCAGCCGTAAGTCTTGATCTCCTTACCTCTGATTTTGCTGCGCGA
G
```

Multiple sequence alignments

Reverse complement alignment

```
c-kit exp CCCTCCTCCCAGCGCCCTCCCTCTGCGCGCCGGCCACGCCCCTCCTCGCCTCCCCTCCCT 60
c-kit-wt  CCCTCCTCCCAGCGCCCTCCCTCTGCGCGCCGGCCACGCCCCTCCTCGCCTCCCCTCCCT 60
c-kit-mut CCCTCCTCCCAGCGAACTCCCTCTGCGCGCCGGCCACGCCCCTCATCGACTCCCCTCCCT 60
          **************..****************************.***.***********
c-kit exp CGCGCCCGCCCG  72
c-kit-wt  CGCGCCCGCCCG  72
c-kit-mut CGCGCACGCACG  72
          *****.***.**
```

Forward alignment

```
c-kit exp CGGGCGGGCGCGAGGGAGGGGAGGCGAGGAGGGGCGTGGCCGGCGCGCAGAGGGAGGGCG 60
c-kit-wt  ---------------------AGGCGAGGAGGGGCGTGGCCGGCGCGCAGAGGGAGGGCG 39
c-kit-mut ---------------------AGTCGATGAGGGGCGTGGCCGGCGCGCAGAGGGAGTTCG 39
                               **.***.****************************..**
orig      CTGGGAGGAGGG  72
c-kit-wt  CTGGGAGGAGGG  51
c-kit-mut CTGGGAGGAGGG  51
          ************
```

In yellow, the point mutations present in the c-kit-mut construct are highlighted. The forward alignment is a partial additional confirmation of the result obtained with the reverse complement alignment, even if it does not cover all the sequence. The results assure the accuracy of the three G4 sequences in both c-kit-wt and c-kit-mut.

c-kit-wt

$^{-158}$CGGGCGGGCGCGAGGGAGGGGAGGCGAGGAGGGGCGTGGCCGGCGCGCAGAGGGAGGGCGCTGGGAGGAGGG^{-87}

c-kit-mut

$^{-158}$CG**T**GCG**T**GCGCGAGGGAGGGGAG**T**CGA**T**GAGGGGCGTGGCCGGCGCGCAGAGGGAG**TT**CGCTGGGAGGAGGG^{-87}

Figure S1. DNA sequences of the two core constructs comprised between position -158 and -87 upstream the Transcription Starting Site (TSS) of the c-kit proximal promoter. Above, the 72 bases of *c-kit-wt* are reported, the sequence of kit2, kit* and kit1 are highlighted in yellow, cyan and green respectively. Below, the G to T point mutations present in *c-kit-mut,* which impair the folding of the three G4, are indicated in red.

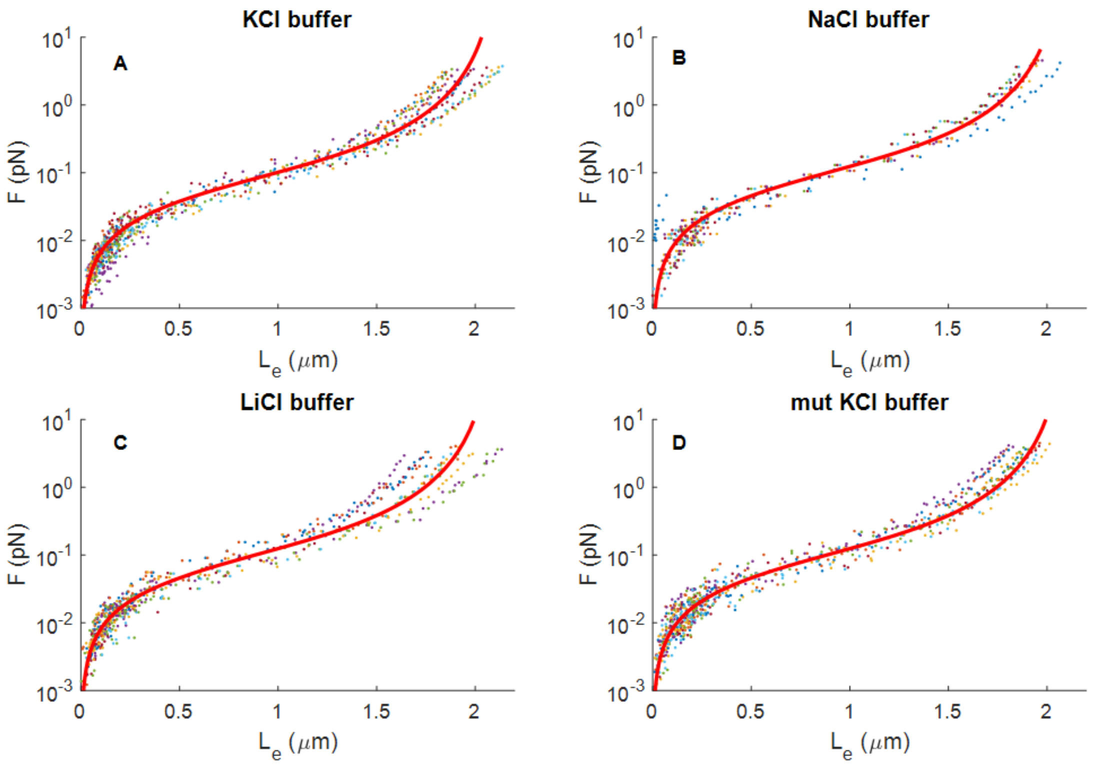

Figure S2. Representative force-extension data obtained with MTs techniques at n_t=0 for the *c-kit-wt* construct in 150 mM KCl (A), NaCl (B) and LiCl (C) buffers and for the *c-kit-mut* construct in 150 mM KCl buffer (D). The colored dots represent the experimental data and the red line represents the WLC fit. The corresponding fitting parameters are reported in Table S1.

	c-kit-wt 150mM KCl	*c-kit-wt* 150mM NaCl	*c-kit-wt* 150mM LiCl	*c-kit-mut* 150mM KCl
L_0 (µm)	2.00 ± 0.12	1.97 ± 0.14	2.10 ± 0.17	2.10 ± 0.11
L_P (nm)	46 ± 4	51 ± 5	46 ± 6	46 ± 3

Table S1. End-to-end length L_0 and persistence length L_P parameters resulting from fitting of the force-extension data acquired for the specified construct to the WLC model in different buffers.

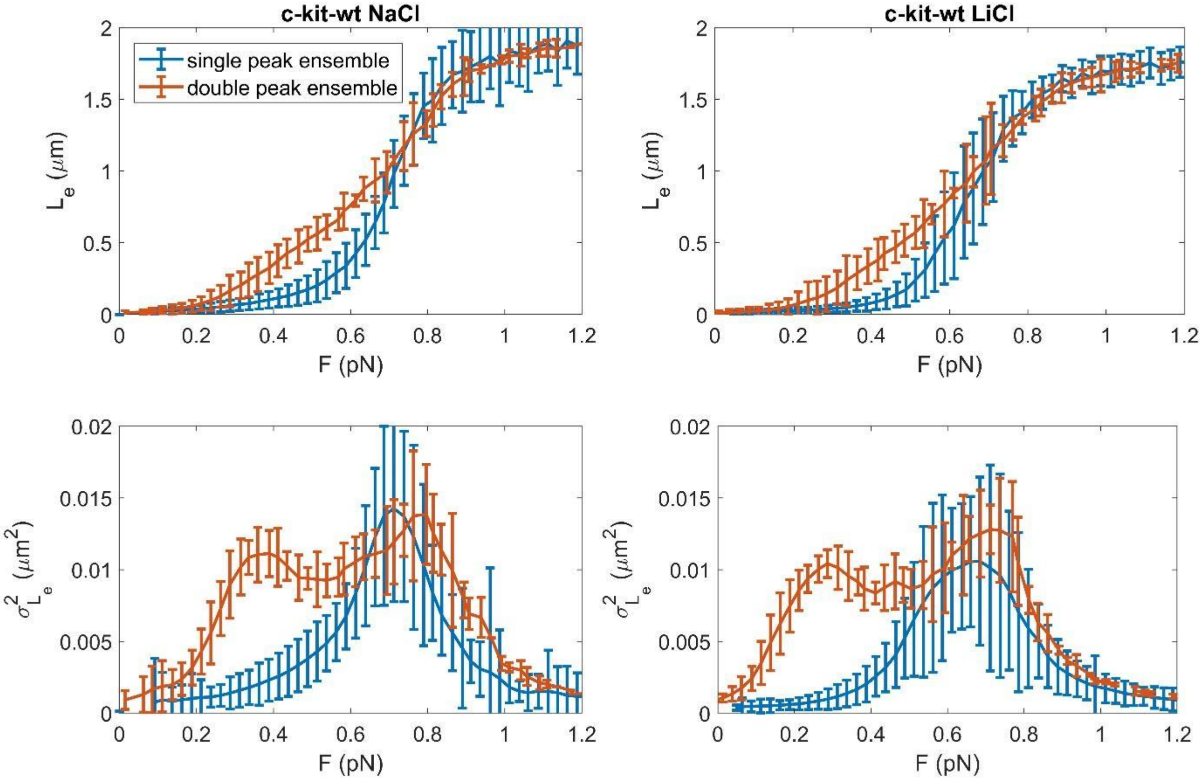

Figure S3. DNA extension (L_e) (upper panels) and corresponding variance ($\sigma^2_{L_e}$) (lower panels) measured at negative imposed turns, n_t=-40, as a function of the applied force (F). Data acquired for c-kit-wt and c-kit-mut in different buffers (150mM NaCl and 150mM LiCl) as indicated by the upper labels.

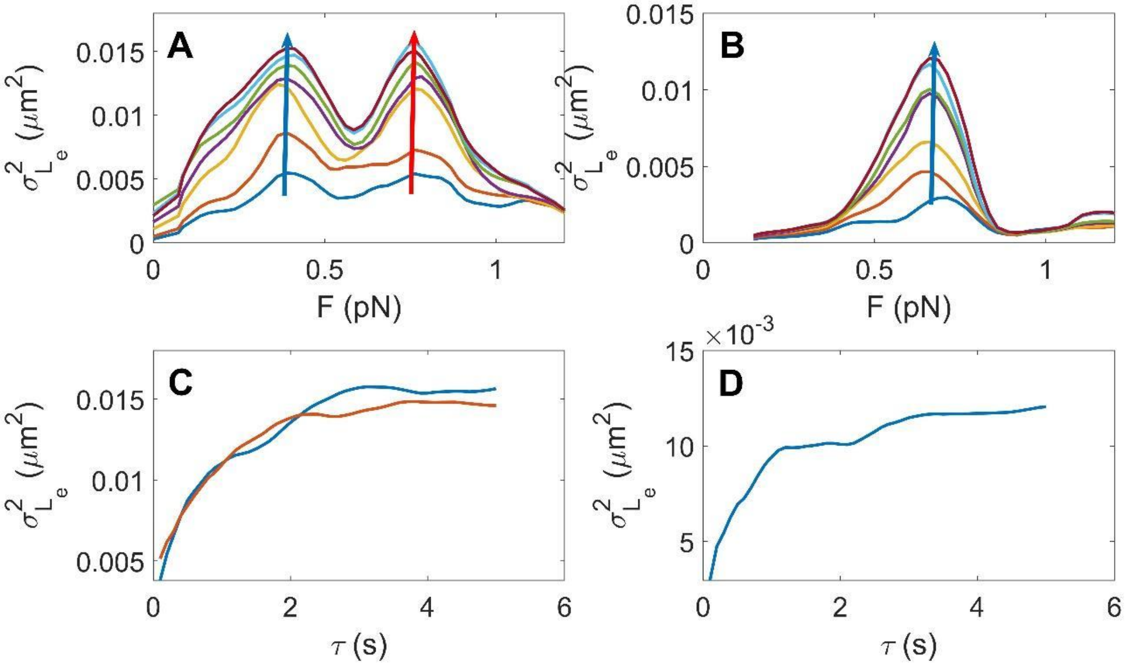

Figure S4. Panels A and B: DNA variance $\sigma^2_{L_e}$ measured as a function of the applied force F for increasing values of temporal windows τ (τ= 0.1; 0.2; 0.5; 1; 2; 4; 5 (s) from bottom to upper curves) used for data acquisition. Measurements acquired for c-kit-wt in KCl and imposed turns n_t=-40 or supercoiling density σ=-0.07. The vertical arrows indicate the force values corresponding to the σ^2_{Le}peaks. The σ^2_{Le} values measured at such forces are reported as a function of tau in panel C and D. Panels C and D: corresponding DNA variance $\sigma^2_{L_e}$ reported at F= 0.39 pN and 0.75 pN (panel C) and F= 0.67 pN (panel D) as a function of the temporal windows τ.

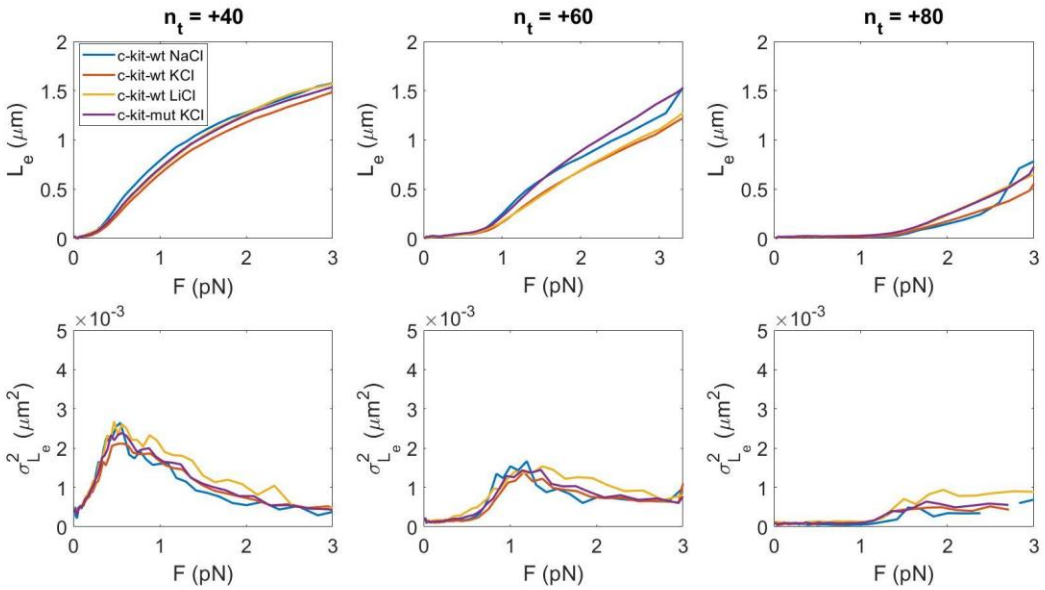

Figure S5. DNA extension (L_e) (upper panels) and corresponding variance ($\sigma^2_{L_e}$) (lower panels) measured for positive imposed turns, n_t, as a function of the applied force (F). Data obtained for three different positive nt (nt= +40, +60, +80, first, second and third column, respectively) for c-kit-wt and c-kit-mut in different conditions (in the presence of 150 mM NaCl, 150 mM KCl or 150 mM LiCl), as indicated by the color code specified in the upper left inset.
